# Sos1 Modulates Extracellular Matrix Synthesis, Proliferation, and Migration in Fibroblasts

**DOI:** 10.3389/fphys.2021.645044

**Published:** 2021-04-06

**Authors:** Isabel Fuentes-Calvo, Carlos Martinez-Salgado

**Affiliations:** ^1^Institute of Biomedical Research of Salamanca (IBSAL), Salamanca, Spain; ^2^Translational Research on Renal and Cardiovascular Diseases (TRECARD)-REDINREN (ISCIII), Department of Physiology and Pharmacology, University of Salamanca, Salamanca, Spain

**Keywords:** Sos1, fibrosis, proliferation, migration, fibroblasts, extracellular matrix synthesis (ECM), ERK, Akt

## Abstract

Non-reversible fibrosis is common in various diseases such as chronic renal failure, liver cirrhosis, chronic pancreatitis, pulmonary fibrosis, rheumatoid arthritis and atherosclerosis. Transforming growth factor beta 1 (TGF-β1) is involved in virtually all types of fibrosis. We previously described the involvement of Ras GTPase isoforms in the regulation of TGF-β1-induced fibrosis. The guanine nucleotide exchange factor Son of Sevenless (Sos) is the main Ras activator, but the role of the ubiquitously expressed Sos1 in the development of fibrosis has not been studied. For this purpose, we isolated and cultured Sos1 knock-out (KO) mouse embryonic fibroblasts, the main extracellular matrix proteins (ECM)-producing cells, and we analyzed ECM synthesis, cell proliferation and migration in the absence of Sos1, as well as the role of the main Sos1-Ras effectors, Erk1/2 and Akt, in these processes. The absence of Sos1 increases collagen I expression (through the PI3K-Akt signaling pathway), total collagen proteins, and slightly increases fibronectin expression; Sos1 regulates fibroblast proliferation through both PI3K-Akt and Raf-Erk pathways, and Sos1-PI3K-Akt signaling regulates fibroblast migration. These study shows that Sos1 regulates ECM synthesis and migration (through Ras-PI3K-Akt) and proliferation (through Ras-PI3K-Akt and Ras-Raf-Erk) in fibroblasts, and describe for the first time the role of the Sos1-Ras signaling axis in the regulation of cellular processes involved in the development of fibrosis.

## Introduction

In most cases, when organs suffer injuries motivated by different disorders or diseases, a complex cascade of cellular and molecular responses triggering fibrosis of the tissue begins. When this phenomenon occurs over a prolonged period of time, this ends up causing irreversible parenchymal damage, cellular dysfunction and functional failure of the organ (Rockey et al., 2015). This process is common in many diseases such as chronic renal failure, liver cirrhosis, chronic pancreatitis, pulmonary fibrosis, rheumatoid arthritis, and atherosclerosis. In almost all cases, fibrosis is not reversible, and therefore the only possible treatments in specific cases are substitution therapies (transplantation). On the other hand, the fact that so many different diseases cause fibrotic processes suggests that most of them share pathogenic pathways. One of the most relevant intracellular pathways involved in virtually all types of fibrosis is that of transforming growth factor beta (TGF-β) (Rockey et al., 2015).

Previous studies of our research group have described the involvement of Ras GTPase isoforms in the regulation of TGF-β1-induced fibrosis. Thus, both K-Ras, N-Ras and H-Ras regulate extracellular matrix (ECM) synthesis, proliferation and migration in fibroblasts (Martínez-Salgado et al., 2006; Fuentes-Calvo et al., 2012, 2013; Muñoz-Félix et al., 2016). We have also observed that deletion of H-Ras reduces renal fibrosis and myofibroblast activation in a fibrotic *in vivo* model induced by ureteral obstruction in mice (Grande et al., 2010). Activation of Ras and its effectors Erk and/or Akt mediates certain pathological effects of the molecules involved in renal fibrogenesis and chronic renal disease, as we reviewed in Martínez-Salgado et al. (2008). On the other hand, Ras participates in the regulation of fibrosis activated by other mediators. Thus, we have also found that TNF-related weak inducer of apoptosis (TWEAK) promotes kidney fibrosis and Ras-dependent proliferation of cultured renal fibroblasts (Ucero et al., 2013).

Son of Sevenless (Sos) proteins are the most widely expressed and functionally relevant family of Ras guanine nucleotide exchange factors (GEFs). There are 2 members in mammals, Sos1 (ubiquitously expressed) and Sos2 (Suire et al., 2019). Sos binds to Ras promoting the release of guanosine diphosphate (GDP) and the subsequent Ras activation after binding to guanosine triphosphate (GTP) (Quilliam et al., 2002). The location of Sos in the plasma membrane is necessary and sufficient for Ras activation (Innocenti et al., 2002). For that purpose, the Src homology 2 and 3 (SH2, SH3) domain–containing adaptor protein growth factor receptor–bound protein 2 (Grb2) recruits Sos to activated growth factor receptors after binding to its C-terminal region (Buday and Downward, 1993). Sos can also be activated by GTP-Ras in a positive feedback mechanism (Margarit et al., 2003). Sos1 and Ras mechanistically mediates kindling-2-induced fibrosis in human kidney tubular epithelial cells (Wei et al., 2014). Moreover, Grb2 and Sos downstream signaling pathways are essential for cardiac fibrosis regulation (Zhang et al., 2003).

Most of studies have been focused on identifying Sos1 functional roles, since the Sos2 isoform seems to be mostly dispensable (Esteban et al., 2000; Arai et al., 2009). However, the role of Sos1 in cellular processes involved in the development of fibrosis has not been studied in detail, nor is the implication of the Ras-mediated main intracellular pathways, Raf-Erk1/2 and PI3K-Akt known in these processes. For this purpose, we isolated and cultured Sos1 knock-out (KO) mouse embryonic fibroblasts (MEFs), the main ECM-producing cells, and we analyzed ECM synthesis and cell proliferation and migration in the absence of Sos1, as well as the role of Erk1/2 and Akt in these processes.

## Materials and Methods

### Cell Culture and Stimulation

**FIGURE 1:**
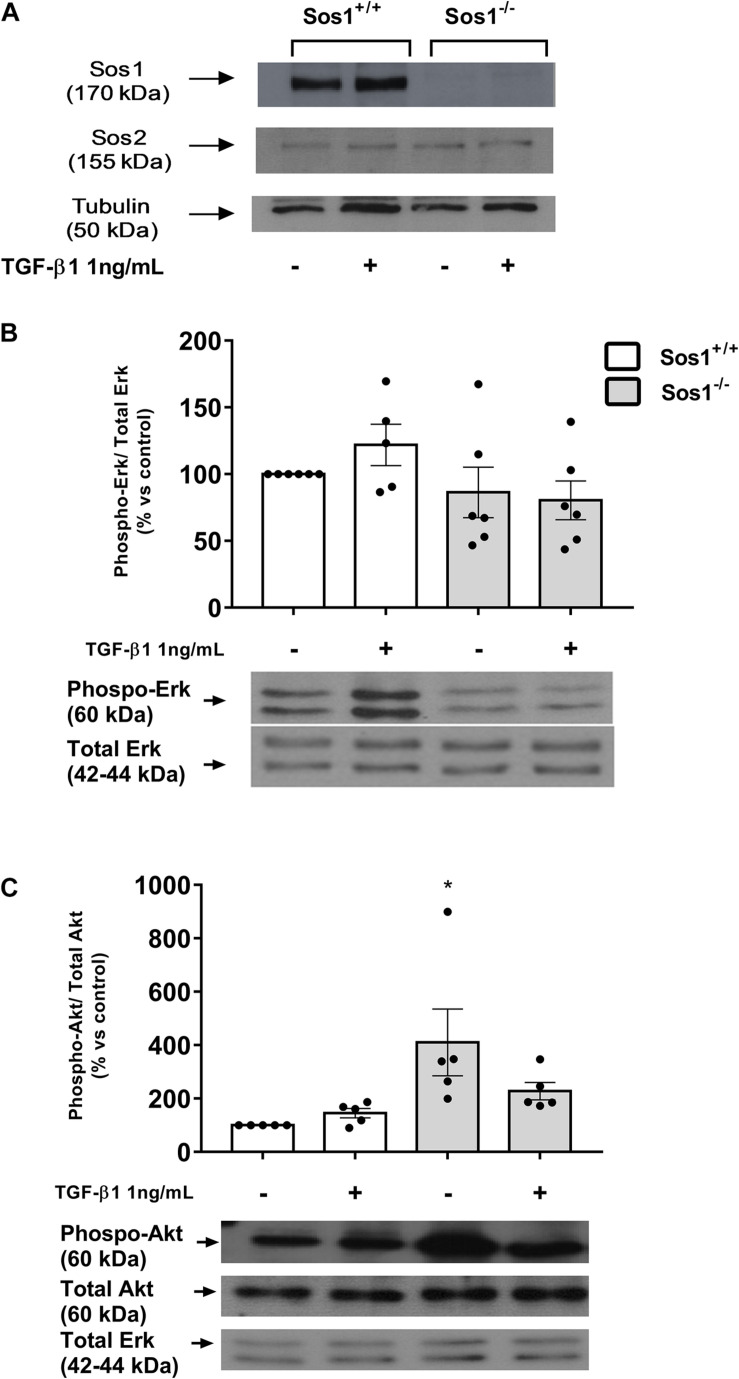
Effect of Sos-1 deficiency on phospho-Erk and phospho-Akt expression in fibroblasts. Sos-1 and Sos-2 expression evaluated by western blot **(A)**; phospho-Erk and total Erk **(B)**, and phospho-Akt and total Akt **(C)** protein expression evaluated by western-blot 24 h after depletion and after TGF-β1 treatment (1 ng/mL, 24 h). Lower panel in panels **(B,C)** shows a representative blot of 5-6 experiments. Histograms in panels **(B,C)** represent the mean ± S.E.M. of the optical density of the bands of 5 experiments, expressed as percentage over basal values (Sos-1^+/+^ fibroblasts in basal conditions, 100%). Histograms correspond to the ratio phospho-Erk 1/2/Erk 1/2 or phospho-Akt/Akt/Erk 1/2 (Erk 1/2 used as loading control). Statistically significant differences: *p < 0,05 vs. Sos1^+/+^ fibroblasts in basal conditions.

Mouse embryonic fibroblasts were subcultured and immortalized from Sos1-KO E13.5 embryos as previously reported (Qian et al., 2000). Fibroblasts were grown at 37°C, 5% CO_2_ in DMEM medium (Gibco-Invitrogen, Grand Island, NY, United States) supplemented with 10% fetal calf serum (FCS, Gibco-Invitrogen) and 100 U/ml penicillin/streptomycin. Cells were seeded in different plastic formats depending on the experiment to be carried out: 100 mm diameter Petri dishes for western blot and 24 well plates for proliferation studies and total collagen measurement. Fibroblasts, after achieving 10-20% (proliferation studies) or 70-80% confluence (studies on extracellular matrix proteins) and serum-starved for 24 h, were treated with human recombinant TGF-β1 (1 ng/mL, R&D Systems Minneapolis, MN, United States) or control vehicle during 24-48 h in serum-free medium. Pharmacological inhibition was performed 30 min before TGF-β1 stimulation with mitogen activated kinase/Erk kinase-1 (MEK-1) inhibitor U0126 (20 μg/mL, Calbiochem-Merck, Madrid, Spain) or the PI3K inhibitor LY294002 (20 μg/mL, Calbiochem-Merck). Figure 1A shows the absence of Sos1 expression in KO fibroblasts.

### Crystal Violet Staining

Total cell number was measured by crystal violet staining as previously described (Fuentes-Calvo et al., 2013). Fibroblasts in 24 well plates were fixed for 10 min with 10% glutaraldehyde, stained for 30 min in 1% crystal violet (Fluka, Buchs, Switzerland) solution and dried overnight. After dissolving with 10% acetic acid, optical absorbance, which was proportional to the number of viable cells in each well, was measured at 595 nm.

### Western Blot Analysis

Protein expression was analyzed by western blot as previously described (Fuentes-Calvo et al., 2013), using the following antibodies: rabbit anti-human Sos1 and rabbit anti-mouse Sos2 (Santa Cruz Biotechnology, Santa Cruz, CA, United States, dilution: 1/1,000), rabbit anti-mouse Akt 1/2 (Santa Cruz Biotechnology, dilution: 1/1,000), rabbit anti-rat Erk1 (Santa Cruz Biotechnology, 1/10,000), mouse anti-human phospho-Erk (Santa Cruz Biotechnology, 1/2,000), rabbit anti-mouse phospho-Akt (Cell Signaling Technology, Danvers, MA, United States, 1/1,000), rabbit anti-mouse collagen type I (Chemicon international, Waltham, MA, United States, 1/20,000) and rabbit anti-mouse fibronectin (Chemicon international, 1/30,000), α-tubulin (Santa Cruz Biotechnology, dilution: 1/1,000). We used total Erk 1/2 levels as loading controls, as their expression does not change in fibroblasts after TGF-β1 treatment, as we had previously found in H-Ras KO, N-Ras KO and H- and N-Ras double KO fibroblasts (Martínez-Salgado et al., 2006; Fuentes-Calvo et al., 2012, 2013; Muñoz-Félix et al., 2016), whereas expression of other frequently used loading controls (actin, tubulin) is modified after TGF-β1 treatment.

### Total Collagen Synthesis Measurement

The incorporation of [^3^H]-proline (American Radiolabelled Chemical, St. Louis, MO, United States) into collagen proteins was used to quantify collagen content in the culture medium, as previously described (Fuentes-Calvo et al., 2013). Radiolabelling was performed incubating 0.15 mM β-aminopropionitrile, 210 mM ascorbic acid, 183 mM proline and 1 μCi/well [^3^H]-Proline (specific activity: 40 Ci/mmol) for 24 h in fresh DMEM serum-free medium. Proteins were precipitated in ice-cold 10% trichloroacetic acid and the pellet was washed and resuspended in 0.1 N NaOH. [^3^H]-Proline incorporated into collagen proteins was measured in a Wallac 1409 DSA β liquid scintillation counter (Perkin Elmer, Waltham, MA, United States).

### Wound-Healing Assay

*In vitro* scratched wounds were created with a straight incision on serum-starved confluent cell monolayers with a sterile disposable pipette tip, as previously described (Fuentes-Calvo et al., 2013). Cell migration into denuded area was monitored over a time course using digital microscopy and cell movement was calculated as the reduction of the wound area over time (in percentage, initial area of the wound: 100%).

### Cell Migration Assay

Fibroblast migration was evaluated with a method based on the Boyden assay as previously described (Muñoz-Félix et al., 2016). Cells were resuspended in 10% FCS DMEM and stained with 2 μM calcein-AM for 15 min in darkness. Cell suspension in 2% FCS medium was loaded into the chamber, and invading cells migrate through and attach to an 8 μm pore size polycarbonate membrane (bottom chamber containing 10% FCS medium), while non-invading cells remain above. Cell migration was analyzed for 24 h after adding the cells in the upper chamber. The number of cells in the bottom of the chamber (migrating cells) was determined by cell fluorescence at λ = 538 each 2 h.

### Statistical Methods

Data are expressed as mean _+_ standard error of the mean (SEM). The Kolmogorov-Smirnov test was used to assess the normality of the data distribution. Comparison of means was performed by two way analysis of variance (ANOVA) and Bonferroni post-test. Statistical analysis was performed using Graph Pad Prism version 5.00 for Windows, Graph Pad Software, San Diego California United States, www.graphpad.com. A “*p*” value lower than 0.05 was considered statistically significant.

## Results

### Increased Akt Expression in Sos1 KO Fibroblasts

We analyzed the changes induced by the absence of Sos1 in the activation of two of the main intracellular pathways stimulated by Ras: rapidly accelerated fibrosarcoma (Raf)-mitogen-activated protein kinase (MAPK) cascade, whose final effect is the phosphorylation (on Tyr204) of extracellular signal-regulated kinases (Erk), and the phosphatidylinositol 3-kinases pathway (PI3K) that leads to phosphorylation (on Ser473) of Akt (protein kinase B). The absence of Sos1 does not affect the expression of phospho-Erk in basal conditions (serum-depleted cells, Figure 1B) but induces a significant increase in the expression of phospho-Akt in these same conditions (Figure 1C).

We stimulated fibroblasts with transforming growth factor beta-1 (TGF-β1), the main cytokine involved in fibrotic processes and in the synthesis of ECM (Leask and Abraham, 2004; Rockey et al., 2015). TGF-β1 (1 ng/mL, 24 h) induces slight increases in Erk and Akt phosphorylation, but those effects disappears completely in the absence of Sos1 (Figures 1B,C). These results indicate that Sos1 is involved in TGF-β1-induced activation of the Raf-Erk and PI3K-Akt pathways, and its absence induces the activation of the PI3K-Akt pathway.

### Increased ECM Proteins Synthesis in Sos1 KO Fibroblasts

**FIGURE 2:**
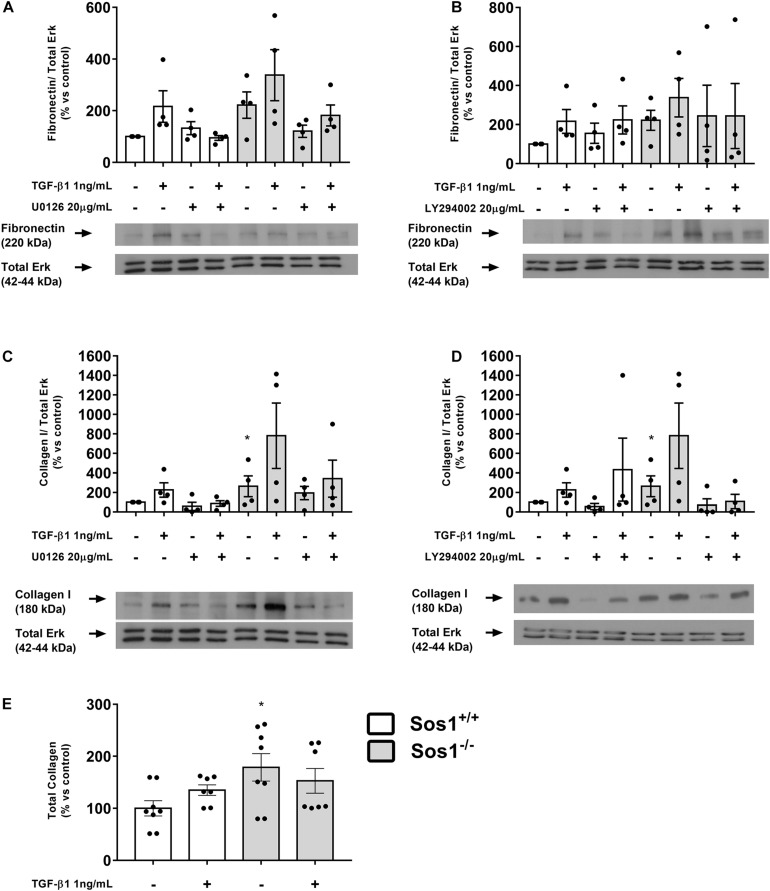
Effect of Sos-1 deficiency and effect of MEK/Erk and PI3K/Akt inhibition on ECM proteins synthesis in fibroblasts. Fibronectin **(A,B)** and collagen type I **(C,D)** protein expression (western blot); effect of phospho-Erk inhibition with 20 μM U0126 **(A,C)** and phospho-Akt inhibition with 20 μM LY294402 **(B,D)** on collagen type I and fibronectin expressions evaluated by western blot; total collagen synthesis **(E)**. Lower panels in **(A–D)** show a representative blot of 4 experiments. Histograms in panel **(A–D)** represent the mean ± S.E.M. of the optical density of the ratio fibronectin or collagen/ERK 1/2 expression (used as loading control), expressed as percentage over basal values (Sos-1^+/+^ fibroblasts in basal conditions, 100%). Histograms in E represent the mean ± S.E.M. of 7-8 experiments expressed as percentage over basal values (Sos-1^+/+^ fibroblasts in basal conditions, 100%), performed in triplicate. Statistically significant differences: *p < 0,05 vs. Sos1^+/+^ in basal conditions.

We assessed the synthesis of ECM proteins by analyzing collagen I and fibronectin expression, as well as total collagen synthesis. The absence of Sos1 induces increases in fibronectin expression (Figures 2A,B), collagen I expression (Figures 2C,D) and in the synthesis of total collagen proteins (Figure 2E). TGF-β1 treatment (1 ng/mL, 24 h) stimulates the expression of fibronectin, collagen I and total collagen synthesis in wild type fibroblasts, but in the absence of Sos1, TGF-β1 does not induce significant increases in fibronectin (Figure 2A,B) and collagen I (Figures 2C,D) expression, nor in the total synthesis of collagen (Figure 2E), because these KO fibroblasts already express high levels of these proteins, probably very close to the maximum synthesis capacity of fibroblasts in culture.

The inhibition of Erk phosphorylation with U0126 (20 μg/mL, 30 min) does not affect the expression of fibronectin or collagen I either in the presence or absence of Sos1 (Figures 2A,C). The inhibition of Akt phosphorylation with LY294002 (20 μg/mL, 30 min) did not induce any noticeable effect on fibronectin expression (Figure 2B) but reduces the expression of collagen I in Sos1 KO fibroblasts, both in basal conditions and after TGF-β1 treatment (Figure 2D). These results show that the PI3K-Akt signaling pathway regulates collagen I expression only in the absence of Sos1.

### Reduced Proliferation in Sos1 KO Fibroblasts

**FIGURE 3:**
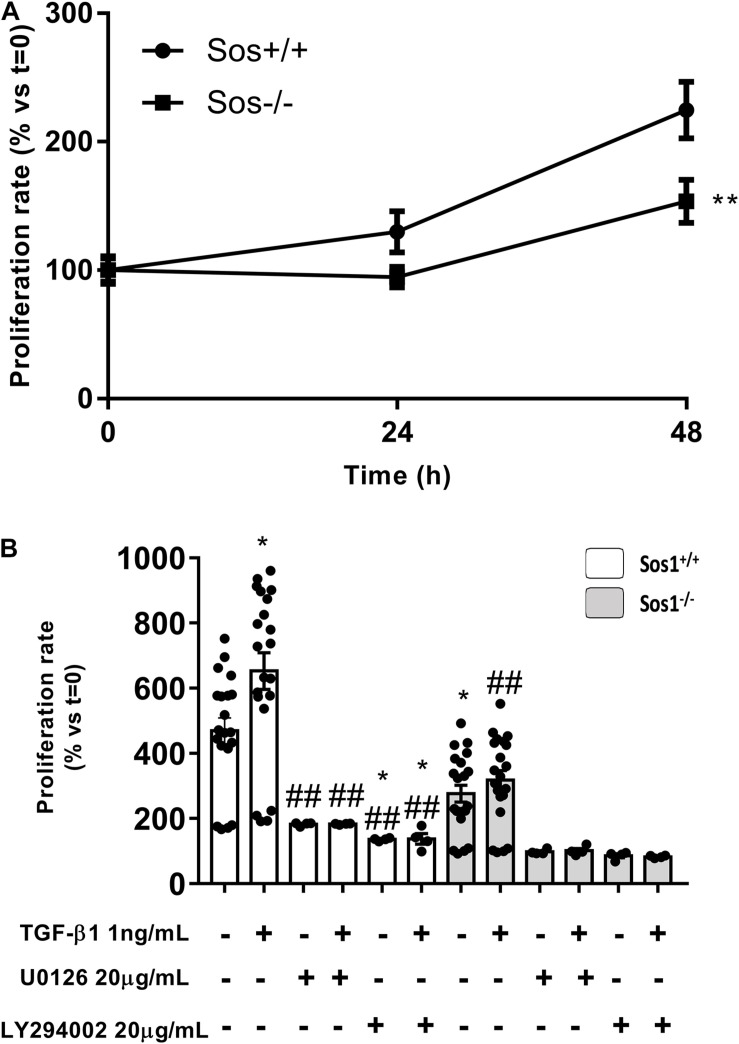
Effect of Sos-1 deficiency and effect of MEK/ERK or PI3K/AKT inhibition on fibroblast proliferation. Cell proliferation measured at 48 h by crystal violet assay **(A)**, expressed as% vs. *t* = 0 (100%); effect of phospho-Erk inhibition with 20 μg/mL U0126 or phospho-Akt inhibition with 20 μg/mL LY294002 on cell proliferation induced by TGF-β1 treatment (1 ng/mL, 48 h), evaluated by crystal violet assay (N = 4-20, in three independent experiments) **(B)**; histogram in B represent the mean ± S.E.M., expressed as percentage over *t* = 0 h (Sos1^+/+^ fibroblasts in basal conditions, 100%). Statistically significant differences: **p < 0,01 vs. Sos1^+/+^ fibroblasts in basal conditions; ^##^*p* < 0,01 vs. Sos1^+/+^ fibroblasts treated with 1 ng/mL TGF-β1.

Cell proliferation is significantly lower in Sos1 KO fibroblasts compared to wild type fibroblasts in basal conditions (serum-depleted cells) as can be seen by a smaller increase in the number of viable cells (assessed by crystal violet nucleus staining) at 24 and 48 h (Figure 3A). In addition, TGF-β1-induced fibroblast proliferation is reduced in the absence of Sos1 (Figure 3B).

In the absence of Sos1, inhibition of Erk phosphorylation with U0126 significantly reduces cell proliferation, both at basal conditions and after TGF-β1 treatment. In addition, pre-treatment with U0126 also inhibits TGF-β1-induced proliferation in wild type fibroblasts. On the other hand, inhibition of Akt phosphorylation with LY294002 also significantly reduces cell proliferation, both at baseline conditions and after TGF-β1 treatment, regardless of the presence of Sos1 (Figure 3B).

All these data suggest that the absence of Sos1 affects fibroblast proliferation, and this process is regulated both by the PI3K-Akt and by the Raf-Erk pathway, the latter signaling pathway being dependent on the presence of Sos1.

### Reduced Migration in Sos1 KO Fibroblasts

**FIGURE 4:**
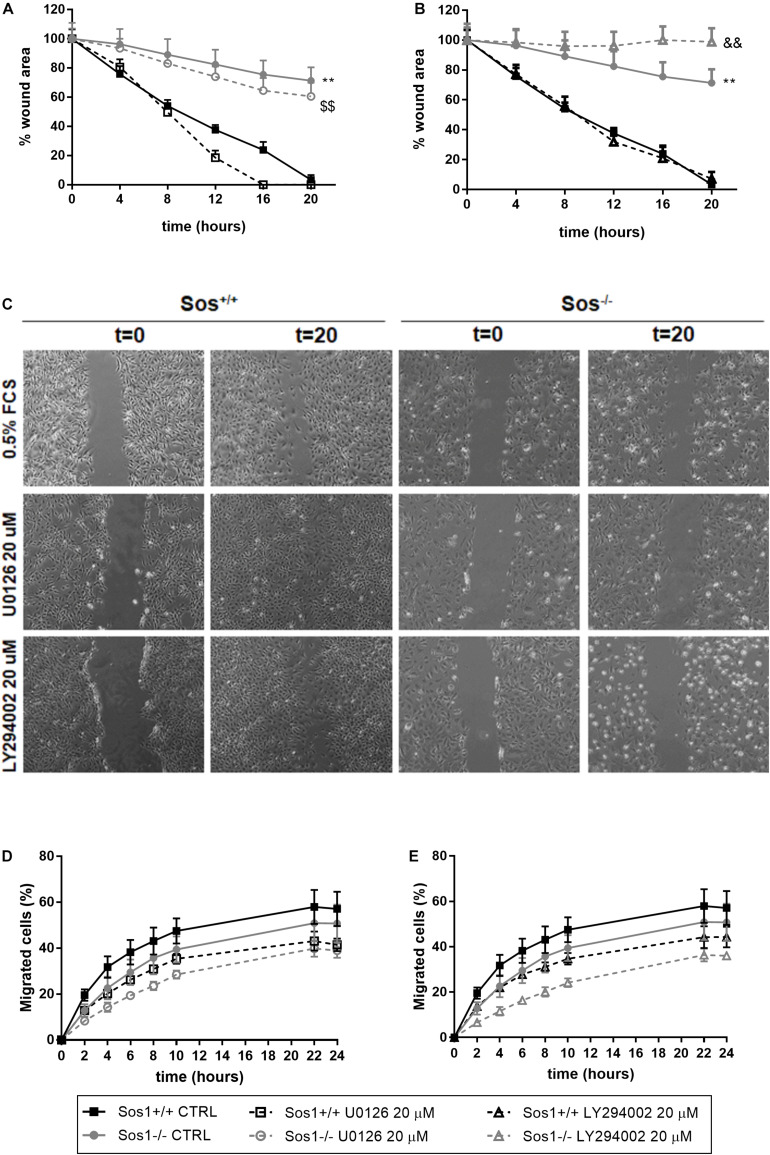
Effect of Sos-1 deficiency on fibroblast migration. Analysis of cell mobility measuring wound closure area **(A–C)** and migration through transwells in a Boyden chamber **(D,E)**, and effect of MEK/Erk inhibition with 20 μg/mL U0126 **(A,D)** or PI3K/Akt inhibition with 20 μg/mL U0126 **(B,E)** in Sos-1 KO fibroblast migration; **(C)** shows representative images (50x) of wound closure at 0 and 20 h. Curve graphs in panels **(A,B)** represent the mean ± S.E.M. of 7 experiments of the time-course reduction of wound area (initial scratched area: 100%). Curve graphs in panels **(D,E)** represent mean ± S.E.M. of 6 experiments quantifying the migrated calcein-AM fluorescent cells expressed as percentage vs. total number of cells (*t* = 24 h, 100%). Statistically significant differences: **p < 0,01 vs. Sos1^+/+^ fibroblasts in basal conditions; $$*p* < 0,01 vs. Sos1^+/+^ fibroblasts treated with U0126; &&*p* < 0,01 vs. Sos1^+/+^ fibroblasts treated with LY294002.

To analyze the involvement of Sos1 in cell migration, we have performed two *in vitro* procedures: scratch time-course assay (Figures 4A-C) and migration chamber assay (Figures 4D,E). The absence of Sos1 impairs fibroblasts migration, as the reduction of the scratched area and the emitting fluorescence of migrated cells is always slower in *Sos1*^–/–^ than in wild type fibroblasts.

Inhibition of Erk phosphorylation with U0126 does not seem to have any effect on the reduction of the scratched area, regardless of the presence of Sos1 (Figures 4A,C). However, in the absence of Sos1, inhibition of Akt phosphorylation with LY294002 reduces the time of closure of the scratched area (Figures 4B,C). Both the inhibition of Erk phosphorylation and Akt phosphorylation slightly reduces the number of migrated cells, both in the presence and absence of Sos1 (Figures 4D,E).

These data suggest that Sos1 is necessary for fibroblast migration, and that the Akt signaling pathway requires the presence of Sos1 to correctly regulate fibroblast migration.

## Discussion

In the last 14 years our research group has described the mediating role of the p21Ras family isoforms in fibroblast biology (Martínez-Salgado et al., 2006, 2008; Fuentes-Calvo et al., 2012, 2013; Ucero et al., 2013; Muñoz-Félix et al., 2016) and in renal fibrosis *in vivo* (Grande et al., 2010). Other research groups have described the involvement of different members and mediators of the p21Ras family in fibrotic processes. RAS p21 protein activator 1 (RASA1) is involved in miR-223 mediated cardiac fibrosis after myocardial infarction (Liu et al., 2018). The farnesyltransferase-Ras-Erk pathway participates in interstitial fibrosis in the aging heart (Trial et al., 2016). KrasG12D mutation contribute to pancreatic tumor development by promoting fibrosis through increased TGF-β signaling (Krantz et al., 2011; Shields et al., 2013). Silencing K-Ras expression inhibits renal fibrosis *in vivo* (Wang et al., 2012), and inhibition of fibroblast Ras/MEK/ERK signaling in fibroblasts might prevent fibrosis (Stratton et al., 2002).

Although the role of the different isoforms of the p21-Ras family on ECM production and fibrosis has been previously described by our group and others, the present study is the first to describe the role of Sos1, the main GEF of Ras, in the production of ECM proteins and associated proliferation and migration in fibroblasts. We show that the absence of Sos1 increases collagen I expression (through the PI3K-Akt signaling pathway), total collagen proteins, and slightly increases fibronectin expression; the absence of Sos1 influences fibroblast proliferation through both PI3K-Akt and Raf-Erk pathways, and Sos1-PI3K-Akt is necessary for fibroblast migration.

Sos1 and Sos2 isoforms show structural homology and similar expression patterns, but the specific functional properties of both isoforms are not fully known. The Sos2 isoform seems to be expendable, since adult Sos2-null mice are perfectly viable and fertile (Esteban et al., 2000). However, Sos1 null mice die during mid-embryonic development (Qian et al., 2000). Therefore, most functional studies have focused on the analysis of Sos1 functional role.

To date, there is only one study analyzing the role of Sos1 in MEFs with a 4-hydroxytamoxifen (4OHT)-inducible, conditional Sos1-null mutation (Liceras-Boillos et al., 2016). Our data are in agreement with this study which describes a mechanistic link between Sos1 and intracellular mitochondrial oxidative stress and shows the prevalence of Sos1 over Sos2 in the regulation of cellular proliferation, migration and viability. We observed that the absence of Sos1 affects fibroblast proliferation, and this process is regulated both by the PI3K-Akt and by the Raf-Erk pathways, the latter signaling pathway being dependent on the presence of Sos1. Our findings are consistent with previous studies of our research group, which show that TGF-β1-induced fibroblast proliferation is reduced in *H-ras*^–/–^/*N-ras*^–/–^ (Martínez-Salgado et al., 2006), *H-ras*^–/–^ (Fuentes-Calvo et al., 2012), *N-ras*^–/–^ (Fuentes-Calvo et al., 2013) and *K-ras*^–/–^ fibroblasts (Muñoz-Félix et al., 2016) with respect to wild type fibroblasts, and this decrease in proliferation is due to changes in Raf-MAPK and PI3K-Akt intracellular signaling. Our study shows that Sos1 is also necessary for fibroblast migration, and the Akt signaling pathway requires the presence of Sos1 to correctly regulate migration. We previously showed that Ras isoforms, as well as PI3K-Akt signaling, are also indispensable to maintain normal fibroblast motility, which was highly restricted in *H-ras^–/–^* (Fuentes-Calvo et al., 2012), *N-ras^–/–^* (Fuentes-Calvo et al., 2013) and in *K-ras^–/–^* fibroblasts (Muñoz-Félix et al., 2016). The decrease in cell migration seems to be dependent on Sos1 and independent of Sos2, since it has also been described that Sos2-KO MEF cultures exhibited similar wound-closure kinetics than wild type fibroblasts (Liceras-Boillos et al., 2016).

Our results suggest that the absence of Sos1 affects cell proliferation and migration, possibly modifying the activity of the different Ras isoforms and two of their main signaling pathways, PI3K-Akt and Raf-Erk; in a similar way, Sos1-Ras might regulate cell migration, in this case mainly through the PI3K-Akt intracellular pathway. According to our hypothesis, it has been described that mir-155-containing macrophage exosomes inhibit cardiac fibroblasts proliferation by downregulating Sos1 (Wang et al., 2017). The role of Sos1 as a regulator of cell migration has been described in macrophages (Baruzzi et al., 2015), glioblastoma cells (Kapoor and O’Rourke, 2010) breast cancer cells (Lin et al., 2018), peripheral CD4(+) T cells (Guittard et al., 2015), etc.

The absence of Sos1 induces a significant increase in both collagen I expression and in the synthesis of total collagen proteins, as well as a slightly higher expression of fibronectin. This regulating role or Sos1 in ECM synthesis might be directly related with previous findings of our research group, which show that ECM synthesis is increased in basal conditions in *H-ras^–/–^*/*N-ras^–/–^* (Martínez-Salgado et al., 2006), *H-ras^–/–^* (Fuentes-Calvo et al., 2012), *N-ras^–/–^* (Fuentes-Calvo et al., 2013) and *K-ras^–/–^* fibroblasts (Muñoz-Félix et al., 2016). To date, there are no studies in the scientific literature describing the involvement of Sos1 in the synthesis of ECM proteins. It is important to keep in mind that, as indicated above, TGF-β1 does not modify ECM protein expression in *Sos1^–/–^* fibroblasts, probably because the absence of Sos1 causes these fibroblasts to already express very high levels of these proteins, and therefore a profibrotic stimulus does not modify the ECM protein synthesis capacity of these cells, which may be close to their maximum level.

Sos1 is necessary for the regulation of collagen I expression through the PI3K-Akt signaling pathway. The absence of Sos1 induces a significant increase in the expression of phospho-Akt in basal conditions (serum-depleted cells). Moreover, the inhibition of Akt phosphorylation reduces the expression of collagen I in Sos1 KO fibroblasts, both in basal conditions and after TGF-β1 treatment. The increase in Akt phosphorylation observed in the absence of Sos1 is consistent with previous studies showing a significant activation of the PI3K pathway in fibroblasts expressing dominant negative mSos1 constructs 53 (Park et al., 2000). This role of the Akt pathway has been previously described by our research group in the Akt-induced increase in ECM synthesis observed in the absence of H- and N-Ras, and for the fact that the inhibition of Akt activation also inhibits ECM synthesis in *H-ras^–/–^/N-ras^–/–^* fibroblasts (Martínez-Salgado et al., 2006). Similarly, *H-ras^–/–^* (Fuentes-Calvo et al., 2012), *N-Ras^–/–^* (Fuentes-Calvo et al., 2013) and *K-ras^–/–^* fibroblasts (Muñoz-Félix et al., 2016) exhibited a higher basal PI3K/Akt activation than wild type fibroblasts, which was also directly related to the higher level of ECM expression presented by these KO fibroblasts. However, the absence of Sos1 does not influence the regulation of the expression of collagen I and fibronectin by the Raf-Erk signaling pathway. Based on these data and in our previous findings, we can affirm that the Sos1-Ras-PI3K-Akt pathway directly regulates collagen I expression.

The role of Sos1 in the regulation of the synthesis of collagen I, proliferation and cell migration that we describe in this study complements the previously cited studies of our research group that show similar effects in the absence of the H-, N- and K-Ras isoforms. All these data together suggest that the Sos1-Ras-PI3K-Akt and Sos1-Ras-Raf-Erk axes regulate the processes described above to a different extent. These data indicate that the absence of any of these mediators, Sos1 or any of the Ras isoforms, deregulates ECM production, proliferation and fibroblast migration. The presence of the three Ras isoforms seems to be necessary for the regulation of these processes, since the individual absence of each of them exerts quite similar effects, although in the case of H-Ras the regulation of these cellular processes seems to be more dependent on the Sos1-Ras-Raf-Erk pathway (Martínez-Salgado et al., 2006; Fuentes-Calvo et al., 2012). These studies confirm the role of the Sos1-Ras signaling axis in processes different of tumor oncogenesis, such as the regulation of cellular processes directly involved in the development of fibrosis. Although the physiological and pathophysiological regulation of fibrosis has an important pivotal axis in TGF-β1 and its signaling pathways (mainly through Smads signaling), the interaction of TGF-β1 and Ras [reviewed in Martínez-Salgado et al. (2008)] must be taken into account when it comes to understanding these processes.

## Data Availability Statement

The raw data supporting the conclusions of this article will be made available by the authors, without undue reservation.

## Author Contributions

IF-C performed the experimental work and the statistical analysis. CM-S designed the study, conceived the experiments, analyzed the results, coordinated the study, and wrote the manuscript. Both authors reviewed the manuscript.

## Conflict of Interest

The authors declare that the research was conducted in the absence of any commercial or financial relationships that could be construed as a potential conflict of interest.
